# Flow mediated dilation of the brachial artery: an investigation of methods requiring further standardization

**DOI:** 10.1186/1471-2261-7-11

**Published:** 2007-03-21

**Authors:** Alon Peretz, Daniel F Leotta, Jeffrey H Sullivan, Carol A Trenga, Fiona N Sands, Mary R Aulet, Marla Paun, Edward A Gill, Joel D Kaufman

**Affiliations:** 1 Department of Environmental and Occupational Health Sciences, University of Washington, Seattle, Washington, USA; 2 Department of Surgery, (Vascular Surgery) University of Washington, Seattle, Washington, USA; 3 Applied Physics Laboratory, University of Washington, Seattle, Washington, USA; 4 Department of Medicine, Division of Cardiology, University of Washington, Seattle, WA, USA; 5 Department of Medicine, University of Washington, Seattle, WA, USA; 6 University of Washington Occupational and Environmental Medicine Program, Department of Environmental and Occupational Health Sciences, 4225 Roosevelt Way NE, Suite #302, Box 354695 Seattle, WA 98195 USA

## Abstract

**Background:**

In order to establish a consistent method for brachial artery reactivity assessment, we analyzed commonly used approaches to the test and their effects on the magnitude and time-course of flow mediated dilation (FMD), and on test variability and repeatability. As a popular and noninvasive assessment of endothelial function, several different approaches have been employed to measure brachial artery reactivity with B-mode ultrasound. Despite some efforts, there remains a lack of defined normal values and large variability in measurement technique.

**Methods:**

Twenty-six healthy volunteers underwent repeated brachial artery diameter measurements by B-mode ultrasound. Following baseline diameter recordings we assessed endothelium-dependent flow mediated dilation by inflating a blood pressure cuff either on the upper arm (proximal) or on the forearm (distal).

**Results:**

Thirty-seven measures were performed using proximal occlusion and 25 with distal occlusion. Following proximal occlusion relative to distal occlusion, FMD was larger (16.2 ± 1.2% vs. 7.3 ± 0.9%, p < 0.0001) and elongated (107.2 s vs. 67.8 s, *p *= *0.0001*). Measurement of the test repeatability showed that differences between the repeated measures were greater on average when the measurements were done using the proximal method as compared to the distal method (2.4%; 95% CI 0.5–4.3; *p *= *0.013*).

**Conclusion:**

These findings suggest that forearm compression holds statistical advantages over upper arm compression. Added to documented physiological and practical reasons, we propose that future studies should use forearm compression in the assessment of endothelial function.

## Background

Impaired endothelial function is recognized as an early and modulating process in the pathophysiology of atherosclerotic cardiovascular disease[1]. Endothelial function is often quantified by flow-mediated dilation (FMD), which represents the endothelium-dependent relaxation of a conduit artery-typically the brachial artery – due to an increased blood flow. Brachial artery reactivity is a frequently used non-invasive ultrasonographic assessment of FMD that indicates endothelium-dependent response to shear stress[2]. This measure is a marker for increased cardiovascular risk[3], and correlates with impaired endothelium-dependent relaxation in the coronary arteries[4]. Due to lack of a standardized method to measure brachial artery reactivity[5,6], we sought to evaluate the impact of different circulatory occlusion sites (upper arm and forearm occlusion locations) and timing of measurements, in order to establish a consistent method for brachial artery reactivity assessment in a future study of environmental health effects. We assessed the magnitude and temporal characteristics of the response as well as for sources and levels of variability and repeatability of measurements. This study leads to further clarification of impacts of brachial artery reactivity technique and analysis.

## Methods

### Study Participants

Twenty-six volunteers participated in the study. Participants were age 18–49, healthy, normotensive, nonsmokers. The study was approved by the University of Washington Human Subjects Review Committee; subjects provided informed consent for all procedures.

### Study protocols

In the first 19 participants, FMD was measured twice on two separate days, at least two weeks apart. In the remaining seven participants, FMD was measured on two separate days, on two occasions on each day separated by one hour for all individuals.

All measurements of brachial artery diameter and FMD were performed in the morning, in a quiet and dark room and at controlled ambient temperatures between 20°C and 26°C. The electrocardiogram (ECG) was continuously monitored. Female participants were examined during the follicular phase of their menstrual cycle. Studies were conducted after an overnight fast of at least 10 hours (water was permitted), with the subjects supine and after 10 minutes of rest. The subject's right arm was comfortably immobilized in the extending position, allowing for ultrasound scanning of the brachial artery 5–10 cm above the antecubital fossa.

In each examination, recording of vessel images were followed by inflation of a cuff to suprasystolic pressure (40 to 50 mmHg above systolic pressure) for 5 minutes as suggested by findings in Corretti et al[7]. Then the brachial artery diameter was imaged and recorded for 3 minutes. The first 19 participants were randomly assigned to cuff inflation either on the forearm, or upper arm. In the remaining seven participants the cuff was inflated on the forearm on the first day and upper arm on the second day. Due to the location of the occluding cuff, the transducer was positioned in a slightly lower position in the studies conducted with proximal occlusion compared to those with distal occlusions.

### Image acquisition

The brachial artery was scanned in the longitudinal section using an HDI 5000 Ultrasound Instrument (Philips Medical Systems, Bothell, WA) with a 5–12 MHz linear array transducer. One experienced sonographer collected all images. Images were digitized from the video output of the ultrasound machine using a frame grabber under control of custom software on a personal computer. Image acquisition was gated with an ECG signal so that images were captured at end diastole in each cardiac cycle.

### Image analysis

Brachial artery diameter was assessed by a single trained operator using an automated, beat-by-beat image processing software package (Vascular Tools 4.6, Medical Imaging Applications, USA). This analysis technique was previously introduced [8] and independently validated[9,10]. Briefly, the operator defined a vascular region of interest, which was then applied automatically to identify the media-to-media diameters in each frame over time. We used two measures of quality control: we excluded from the analysis frames with a detected vessel border smaller than 70% of the width of the region of interest (confidence index > 70%) and, for post-cuff deflation sessions only, frames with diameters that differ by more than 1 standard deviation from a polynomial fit.

**Figure 1:**
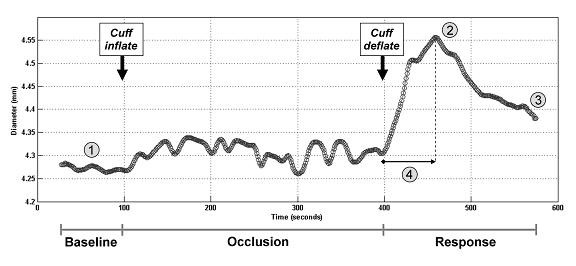
**Representative flow-mediated response of brachial artery (forearm occlusion)**. Measurements are used to characterize the brachial artery response: 1) baseline diameter (D_BL_), 2) maximum diameter after cuff release (D_max_), 3) diameter 3 minutes after cuff release, 4) time of maximum diameter after cuff release.

Custom software developed in the MATLAB programming environment (The MathWorks, Natick, MA) was used to extract measurements from the time series of diameter measurements. The capture time for each image was decoded by optical character recognition of the time of day displayed on each image frame. The sequence of diameter measurements was then interpolated to equally-spaced time samples at 1-second intervals. This interpolated sequence was smoothed by a 9-point median filter followed by a 9-point Gaussian-weighted moving average. The following parameters were measured from the resulting time sequence of brachial artery diameter (Figure 1): mean baseline brachial artery diameter (D_BL_) measured in millimeters; peak FMD measured in percentage and calculated as [(D_max_-D_BL_)/D_BL_] × 100; time-to-peak FMD measured in seconds; diameter change at the end of the post-cuff deflation period (D_end_) measured in percentage and calculated from the average diameter of the last 10 seconds of the post-cuff release period relative to D_BL_.

### Statistical analysis

Descriptive data are presented as mean ± standard error (SE) unless specified otherwise. D_max_, time-to-peak FMD, and D_end _were compared between cuff positions using ANOVA with correction for gender and the level to which the blood pressure cuff was inflated (i.e. 40 or 50 mmHg above systolic blood pressure).

Variability and repeatability of the test were measured and compared between the two cuff positions. Assessment of variability included: calculation of the coefficient of variation (CV) of the baseline diameters from accepted frames to determine the within-session variability; mean CV from 2 or more baseline measurements for each of the first 19 participants as a day-to-day variability. Both descriptors were calculated for the two cuff locations and then compared by independent *t *test.

Repeatability for FMD analysis for each cuff location was calculated using intraclass correlation coefficient (ICC) in a two-way mixed effects model, and Bland & Altman plots with calculation of the repeatability coefficient[11]. SPSS 12.0 (Chicago, Illinois) statistical software and Microsoft Excel were used.

In addition, a linear model was run to assess whether the absolute values of the difference in FMD between test and retest were different using the proximal versus the distal circulatory occlusion. To account for the correlation between distal and proximal comparisons done on same subjects, a generalized estimating equations (GEE) approach was used, assuming an exchangeable correlation structure. S-plus 7.0 (Insightful Corp. 2005) statistical software was used. Results from all tests were considered statistically significant if *p *<*0.05*.

## Results

A total of 62 ultrasound examinations of the brachial artery followed by assessment of endothelium-dependent flow mediated vasodilation were analyzed in this paper, 37 with upper arm cuff location, and 25 with forearm cuff location. Participants' mean age was 28.4 (range, 20–42 yr). More male (N = 16) than female (N = 10) participated in the study.

Overall, mean baseline brachial artery diameter from 37 sessions with upper arm occlusion was smaller (3.8 ± 0.1) than that from 25 sessions acquired with forearm occlusion (4.2 ± 0.2) (*p *= *0.058*). This difference between baseline diameters appears to be related to the slightly different location of the ultrasound transducer on the participants' arm. In fact, similar results were obtained after limiting analysis to those participants that were evaluated with both proximal and distal occlusion (data not shown). Mean number of frames obtained for baseline diameter assessment was similar for both proximal and distal cuff locations (81.7 vs. 81.5 respectively).

### Flow Mediated Dilation

Pooling all the brachial artery reactivity tests together, we found that peak FMD using the upper arm (N = 37) was greater than that for the forearm (N = 25) (16.2 ± 1.2% vs. 7.3 ± 0.9, p < 0.0001). The gender and the pressure to which the cuff was inflated did not alter these results (data not shown).

**Figure 2:**
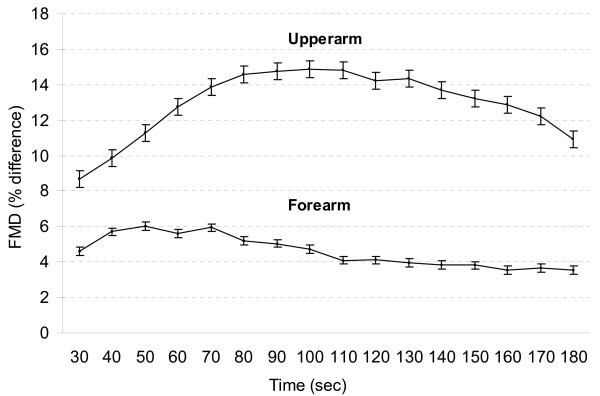
**Time course of brachial artery dilation following reactive hyperemia**. Time course in 10-second intervals of brachial artery FMD expressed as percent difference from baseline diameter after upper arm cuff inflation (upper curve) and forearm cuff inflation (lower curve). Each point in the graphs represents an average of all data available in that time-point; the error bars represent one standard error to the mean.

The time course of the average dilation of the brachial artery from cuff release is shown in figure 2. Upper arm cuff placement resulted in a slow increase, with a peak diameter of 15.9 ± 1.2% after 109 seconds; forearm cuff placement generated a smaller peak (6.6 ± 0.8%) after only 67 seconds post-cuff-deflation. For the entire recording time (180 seconds) brachial artery diameter values were greater than baseline for both cuff locations.

On average, the time-to-peak FMD was 107.2 ± 6 seconds for upper arm cuff inflation and 67.8 ± 8.9 seconds for forearm cuff inflation (*p *= *0.0001*). After peak dilation, the artery diameter decrements were slower with proximal occlusion than distal occlusion. At the end of the three-minute image capture period after cuff release, D_end _was 11.3 ± 1.2% with proximal cuff occlusion, and 3.3 ± 0.8% with the distal cuff occlusion (*p *= *0.0001*).

**Figure 3:**
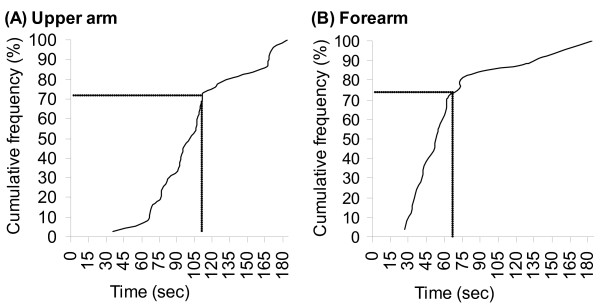
**Cumulative frequency of individual peak dilations after cuff deflation**. (A) Upper arm cuff inflation; (B) Forearm cuff inflation. Dashed lines represent the percentage of individuals with maximal response at the average time-to-peak FMD for each of the cuff locations.

The peak FMD responses from all examinations (N = 62) grouped in a cumulative frequency representation shows that utilizing the proximal arm test, ~35% of subjects had not reached their maximal FMD response by the average time (107.2 seconds) (Figure 3a). For the forearm test, ~30% of individuals had not reached their maximal response by the average time (67.8 seconds) (Figure 3b). For both the proximal and distal tests, all subjects reached their maximal response by 180 seconds.

### Variability and Repeatability

For the 62 studies for which ~80 baseline frames were taken in a single session, the within-session diameter variability was small (CV = 0.85 ± 0.04%); there was no statistically significant difference between the upper arm cuff location (CV= 0.85 ± 0.05%) and the more distal location (CV= 0.84 ± 0.06%). Day-to-day variability of baseline diameter was also small for both the distal cuff placement (CV = 3.4 ± 1.3%) and the proximal one (CV = 5.1 ± 1.7%), with no statistically significant difference between them.

**Figure 4:**
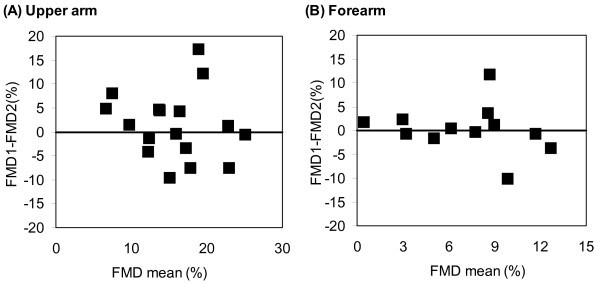
**Reproducibility of Flow-Mediated Dilation (FMD) using Bland-Altman plots**. (A) Upper arm cuff inflation; (B) Forearm cuff inflation. The x-axis illustrates the individual average values of peak-FMD. The y-axis shows the differences between two peak FMD. All values are presented as percentage.

The repeatability of the test for peak FMD using Bland-Altman plots is presented in figure 4. The coefficient of repeatability thus calculated is high for both proximal cuff location (CR = 14.11%) and the distal cuff location (CR = 10.11%).

The intraclass correlation coefficient (ICC) between peak-FMD of two repeated measures was 0.6 (95%CI -0.2:0.8) for the upper arm occlusions, and 0.6 (95%CI -0.7:0.9) for the forearm occlusion. Using the GEE approach we found that the magnitude of the differences between the repeated measures were on average 2.4% (95% CI 0.5–4.3; *p *= *0.013*) greater when the measurements were done using the proximal method as compared to the distal method.

## Discussion

In this study we evaluated statistical reasons to support application of specific conditions to FMD testing on the brachial artery. While assessment of FMD of the brachial artery either with proximal (upper arm), or distal (forearm) occlusion has been found to be correlated with coronary endothelial function[12,13], we evaluated whether other test characteristics may provide a basis for selecting one approach over the other.

We have confirmed the well established finding that the maximal FMD is greater and delayed with upper arm occlusion[6,14-20]. We showed a significantly greater and delayed FMD following proximal occlusion (16.2% and 107.2 seconds, respectively) compared to distal occlusion (7.3% and 67.8 seconds). The difference in the magnitude of FMD between occlusion sites (8.9%) is greater than the difference reported in a recent meta-analysis (2.4%)[18]. This discrepancy may be due to three major reasons. First, the measures were made in populations with different baseline endothelial function. Second, differences in baseline diameters in each of the two occlusion sites, may have affected the shear stress, the stimulus for endothelium-dependent dilation[21]. In this study, shear stress was not analyzed. Finally, our use of an automated method of analysis with all available frames. Others studies that demonstrated greater FMD in response to proximal occlusion rather than distal occlusion [14-20] captured brachial artery images continuously, but then examined frames from specific time-points to define the artery's diameter and the frequency of these intervals was not consistent between studies.

The time to maximal FMD may vary not only by occlusion site, but also by the method used to detect the diameter from captured frames. For example, adhering to the recommended time interval to maximal FMD at 60 seconds after the release of the occlusive cuff [22] would have resulted in missing the true peak-FMD of 40% of the measurements we obtained with forearm occlusion (figure 3b), and about 95% of the cases of upper arm occlusion (figure 3a). Random measurement errors and underestimation of the actual FMD are less likely when examination of brachial artery dilation is done continuously over the entire course of the session and analyzed from all available images with automated computer based edge detection.

The assessment of FMD of the brachial artery is a functional bioassay for *in vivo *endothelial function in humans. It has been stated that the primary mechanism responsible for vessel dilation with forearm occlusion is endothelial release of nitric oxide(NO)[23]. Doshi et al. demonstrated that most of the dilatory response to proximal occlusion is NO-independent[14]. Additional mechanisms, such as direct effect of hypoxia on the smooth muscle, altered myogenic response, or altered release of other mediators involved in vessel reactivity, may be involved in the dilation of the brachial artery following upper arm occlusion. NO-mediated vasodilation occurs ~60–80 seconds following reperfusion[24,25]. Therefore, in the absence of a pharmacologic intervention, the increased time to maximal FMD and the prolonged dilatory response (D_end _11.3% vs. 3.3% *p *<*0.0001*) for proximal occlusion relative to distal occlusion may reflect other underlying mechanisms. NO is not the only mediator involved in FMD following proximal and distal occlusions; other endothelium-derived factors are probably involved differently with the two occlusion locations. It is essential to conduct validation studies in order to understand the role of different components of endothelial function in one approach or the other of the test.

Many studies have assessed the variability of the brachial artery diameter measurements, both at baseline and during the dilatory response. While the baseline diameter was shown to have relatively small variability (CV range 1–13%), the maximal FMD was associated with a much greater range of variability (CV range 1–84%)[26]. Various factors may contribute to the variability of brachial artery diameter, and these can be largely divided into technical and physiological factors[27]. Ultrasound of the brachial artery is technically challenging, and measurement errors – even in magnitude of fraction of millimeters – especially when added to effect of intrinsic factors affecting FMD, may affect the accuracy of the test. Therefore, it is essential to apply a rigorous methodology to the test. In doing so, we utilized a single ultrasound machine, all studies were performed by one technician, and all images were read by one off-line reader. We found relatively low within-session variability and day-to-day variability of baseline diameter with no difference between proximal and distal placements. Similar day-to-day variability was found by Uehata et al[28]. Greater variability of peak FMD is shown by the relatively high fraction of participants (more than a third, in both occlusion sites) that did not reach their maximal FMD by the average time-to-peak FMD (figure 3). The small impact of technical factors on baseline brachial artery diameter variability may indicate that physiological factors contribute most of peak FMD variability as suggested previously[29].

In this study we did not quantify the shear stress. It has been observed that the shear stress computed as area under the curve, is the major contributor to the magnitude of FMD[30]. Therefore it is plausible that variability in shear stress may affect variability and repeatability of FMD, and understanding this interplay requires additional study.

We also assessed the repeatability of FMD, an important quality of this outcome as a potential tool in clinical studies where repeated measures are needed, and as a potential indicator for the risk of future coronary disease. Previous studies have shown inconsistent results of brachial artery FMD repeatability. Malik at al[31] reported poor repeatability of FMD with an intraclass correlation coefficient of 0.1. In contrast, Welsch et al[32] reported an intraclass correlation coefficient of 0.92 comparing day-to-day FMD. We demonstrated a reasonable repeatability of FMD measurements. Repeatability of FMD was better for the distal cuff location as shown by the smaller coefficient of repeatability (CR = 10.1% vs. 14.1%) and the greater difference on average of two repeated measurements with upper arm cuff placement (2.4%). Even with accurate methodology, our repeatability was only modest; suggesting that some degree of measurement error is inevitable in any setting. However, it is reasonable to assume that studies that use more than one sonographer or are performed in more than one center may be subject to a greater degree of measurement error than observed in this study. Hence, such studies must be extremely well coordinated, using highly trained technical staff, and probably employ a larger sample size than single site studies. The use of duplicate measurements may be important in reducing measurement error, as evidence show that repetitive reactive hyperemia does not effect FMD measurements [33], though the minimum time between measurements has not been clearly identified.

## Conclusion

For brachial artery reactivity to be a widespread clinical and research tool, the flow mediated dilation measurement of the brachial artery should be standardized, and centers should adhere to a more consistent analysis protocol. Our data suggest that the choice of the location of cuff inflation may have statistical implications that favor the forearm approach. There are established physiological reasons that support employing one approach over the other. While FMD following distal occlusion is mainly NO dependent, other – not as well elucidated – factors are involved in the dilatory response to upper arm occlusion. In future studies we will study the effects of an environmental insult on FMD of the brachial artery, while measuring different components of endothelial function. In the absence of a well characterized mechanism involved in the flow mediated dilation of the brachial artery with upper arm occlusion, based on our results, we suggest that the forearm occlusion be used. The cuff should be inflated five cm distal to the elbow for five minutes, then, in the post-hyperemic phase of the test the brachial artery should be scanned continuously. Post-cuff deflation examination should be continued for three minutes in healthy adults, whereas, in adults at risk for CAD or with known disease, it is likely necessary to capture the artery's images for longer periods. Last, we suggest also that the images be analyzed with an automated computer-based edge detection algorithm. This will allow for a time series analysis of all brachial artery diameters and identification of the true maximal FMD. More studies performed with standardized method are needed to confirm our findings and allow for potential future use of the test for clinical purposes.

## Competing interests

The author(s) declare that they have no competing interests.

## Authors' contributions

AP – participated in data collection, performed analysis and interpretation of data and drafting of the manuscript. DL – participated in analysis of data and drafting of the manuscript. JS – contributed to conceive the study and its design and participated in interpretation of data. CT – participated in the design of the study and its coordination. FS – participated in data collection and analysis. MA – carried out coordination of the study. MP – participated in the design of the study and data collection. EG – participated in the design of the study and interpretation of data. JK – conceived the study and participated in its design and interpretation of data. All authors read and approved the final manuscript.

## Pre-publication history

The pre-publication history for this paper can be accessed here:
